# Sunlight-Driven Photochemical Removal of Polypropylene Microplastics from Surface Waters Follows Linear Kinetics and Does Not Result in Fragmentation

**DOI:** 10.1021/acs.est.3c07161

**Published:** 2024-03-15

**Authors:** Erin Tuttle, Charlotte Wiman, Samuel Muñoz, Kara Lavender Law, Aron Stubbins

**Affiliations:** † Department of Biological and Physical Sciences, Assumption University, Worcester, Massachusetts 01609, United States; ‡ Department of Marine and Environmental Science, Northeastern University, Boston, Massachusetts 02115, United States; § Department of Civil and Environmental Engineering, Northeastern University, Boston, Massachusetts 02115, United States; ∥ Sea Education Association, Woods Hole, Massachusetts 02540, United States; ⊥ Department of Chemistry and Chemical Biology, Northeastern University, Boston, Massachusetts 02115, United States

**Keywords:** Microplastics, Photochemistry, Photodissolution, Polypropylene, Fragmentation, Dissolved organic carbon, Size distribution

## Abstract

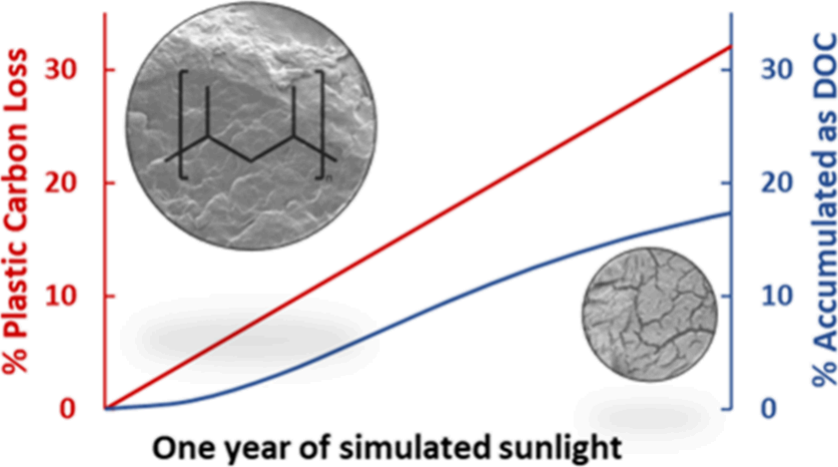

Floating microplastics are susceptible to sunlight-driven photodegradation, which can convert plastic carbon to dissolved organic carbon (DOC) and can facilitate microplastic fragmentation by mechanical forces. To understand the photochemical fate of sub-millimeter buoyant plastics, ∼0.6 mm polypropylene microplastics were photodegraded while tracking plastic mass, carbon, and particle size distributions. Plastic mass loss and carbon loss followed linear kinetics. At most time points DOC accumulation accounted for under 50% of the total plastic carbon lost. DOC accumulation followed sigmoidal kinetics, not the exponential kinetics previously reported for shorter irradiations. Thus, we suggest that estimates of plastic lifespan based on exponential DOC accumulation are inaccurate. Instead, linear plastic-C mass and plastic mass loss kinetics should be used, and these methods result in longer estimates of photochemical lifetimes for plastics in surface waters. Scanning electron microscopy revealed that photoirradiation produced two distinct patterns of cracking on the particles. However, size distribution analyses indicated that fragmentation was minimal. Instead, the initial population of microplastics shrank in size during irradiations, indicating photoirradiation in tranquil waters (i.e., without mechanical forcing) dissolved sub-millimeter plastics without fragmentation.

## Abstract 2

Photochemical losses of sub-millimeter microplastics occurred without production of fragments, indicating photochemistry alone does not fragment polypropylene microplastics. Photochemical losses of microplastic mass followed linear kinetics resulting in longer estimates of plastic lifetimes at sea.

## 1 Introduction

Since the beginning of widespread plastic manufacturing and use in the 1950s, the mass of plastic-carbon (e.g., mass of carbon contained in synthetic polymers, plastic-C) has grown to rival biogeochemically relevant natural carbon stocks.^1^ Much of this plastic is used in the production of one-use or short-lifespan commercial items; of all plastics produced since 1950 only an estimated 30% is still in use.^2^ In 2010 mismanaged waste resulted in an estimated 4.8 to 12.7 million metric tons of plastics entering the ocean, a number estimated to have increased over the subsequent five years.^3,4^ Floating plastic debris in the ocean is considered extremely long-lived, with evidence of persisting at the ocean surface for decades,^5^ and experiencing embrittlement and fragmentation by weathering and wave action.^6−8^ The estimate of buoyant plastic pieces floating on the ocean surface is in the trillions, but may not account for all buoyant plastics believed to have entered the ocean.^9,10^ In the past decade evidence has grown that some ocean plastics are undergoing transport or removal that is not fully understood,^11^ although recently updated models suggest undercounting of large plastic debris may be responsible for the apparent mass imbalance.^12^

Many routes of plastic removal from surface waters have been studied including biofouling, ingestion, fragmentation, nanoparticle production, and photodegradation. Fragmentation is the process of generating multiple smaller particles from a single larger particle. In a system experiencing mechanical forces that induce fragmentation, particle count increases as particle size decreases in a power relationship.^18^ Power function fragmentation models have been used to predict and describe the size distribution of surface ocean plastics, and surveys of aquatic plastic pollution support this model for microplastics larger than ∼5 mm.^19−28^ Production of large numbers of nanoparticles or other fragments too small to capture by current sampling methods (e.g., most studies use tow nets with 200–300 μm screens^1,11^) has also been observed in laboratory experiments.^13−16^ Detection of nanoparticles in ocean samples has not been reported in many studies; however, nanoplastics of mixed polymer type were reported in the North Atlantic.^17^ While mixing into deeper waters,^29^ biofouling induced sinking,^30−33^ and ingestion^34−36^ offer routes of sub-millimeter plastic transport, fragmentation may result in production of undersampled microplastics or nanoplastics, and photodegradation is a compelling mechanism for sub-millimeter plastic removal.

In the presence of sunlight, water, and oxygen, plastics photodegrade, accumulating oxidative products on the plastic’s surface, releasing carbonaceous gases (e.g., carbon dioxide and carbon monoxide), volatiles, and dissolved organic carbon (DOC).^37−39^ The pure polymeric structure of common polyolefins, polyethylene (PE), and polypropylene (PP) does not absorb environmentally relevant wavelengths of ultraviolet light and therefore should not be inherently chemically reactive to sunlight, yet, these plastics photodegrade in seawater.^37,40,41^ This may be due to the presence of additives or structural abnormalities^42^ that can initiate radical reactions^43^ which then propagate along the polymer chains resulting in oxidation and chain scission.^8,44^ The accumulation of DOC as PP photodegrades has been reported to follow exponential kinetics (Zhu et al).^41^ Based on this finding, it was suggested that plastic mass loss may also follow exponential kinetics; however, in this earlier work only the initial and final plastic masses of a short irradiation were measured. A progressive time series tracking PP mass loss kinetics over long-term irradiation is needed to fully capture PP mass loss kinetics.

Plastic photodegradation increases oxidation and crystallinity and decreases molecular weight, primarily impacting the first ∼100 μm surface layer of plastics.^45^ Crack formation also begins on the particle surface, permeating on average 10 to 100 μm deep.^46^ Thus, we hypothesize that because photodegradation predominantly involves chemical alterations and product formation at the particle surface, over time the size of the initial plastic particle will decrease as plastic-C is converted to other forms such as DOC and carbon dioxide, serving as a nonfragmenting removal mechanism for small plastic particles at the ocean surface. While fragmentation of PP during irradiation has been reported, these experiments were conducted dry^14^ or in turbulent water.^47^ Whether photochemistry alone can fragment floating plastics into microplastics with the power-law size distribution, without concurrent mechanical force, is not well understood.

To improve understanding of the fate of microplastics in sunlit waters, including the kinetics of photochemical dissolution, the fate of plastic-C, and photofragmentation in the absence of mechanical forces, sub-millimeter PP microplastics were irradiated in a solar simulator for 364 days. PP was used as it is prevalent in surface ocean samples,^48^ is inherently buoyant due to a density lower than seawater (seawater ∼1.05 g cm^–3^, PP 0.83–0.85 g cm^–3^), and photodegrades faster than PE, the other most abundant, buoyant, marine microplastic.^41^ PP standards rather than postconsumer or field collected PP were used to capture the full progression of degradation beginning with unweathered, well characterized (i.e., a standard) plastic and to allow others to repeat or expand upon this study using the same polymer sample. Future work investigating other polymer types and formulations of PP will be needed. Irradiations were conducted in ultrapure water which lacks reactive ions present in aquatic ecosystems, as ultrapure water also lacks organic carbon sources found in natural waters, allowing better resolution of the DOC and any organic particles (i.e., nanoplastics) formed as photodegradation products.^41^

During the irradiation, time series data for plastic mass loss, plastic-C loss, DOC accumulation, and microplastic particle size distribution were collected. Scanning electron microscopy (SEM) provided additional insight into the physical breakdown of the microplastics. The data are presented and used to test the hypothesis that photochemistry without mechanical forces does not fragment plastics but instead leads to a gradual reduction in the size of the initial population of microplastics. Data were also used to assess the kinetics of both DOC accumulation and plastic losses (as total mass and organic carbon mass of all particulate matter), offering insight into the fate of PP derived carbon.

## 2 Materials and Methods

### 2.1 General Sample Handling

To avoid plastic and carbon contamination, all quartz and glassware was cleaned by soaking overnight in a pH 2 hydrochloric acid bath, rinsed five times with ultrapure water (Milli-Q, Millipore), dried overnight at 60 °C, and then combusted at 500 °C for 5 h to remove trace organic carbon. A glass vacuum filtration setup was used to filter particulates onto similarly precombusted 47 mm Whatman GF/F glass microfiber filters (nominal pore size 0.7 μm).

### 2.2 Plastic Preparation and Irradiation

Isotactic PP granules (average *M*_w_ = 340,000; average *M*_n_ = 97,000; Aldrich, 427861) were used to generate microplastics. Attenuated total reflectance Fourier transform infrared (FT-IR) spectroscopy characterization of this plastic was conducted by the manufacturer and is available online. The PP granules were submerged in liquid nitrogen for 30 s and then transferred to a spice grinder and ground for 1 min in 5 s pulses. The PP was not evaluated for thermal oxidation following the cryogen cooled grinding procedure and any chemical changes would have impacted all time points and the dark controls as particles were prepared as a single batch. After grinding, the PP was dry sieved with a sieve shaker plate (Gilson SS-3) to obtain a 300 to 600 μm size class. The freezing and grinding process was repeated for particles larger than 600 μm. Once prepared, the PP particles were wet sieved to remove plastic dust and other smaller particles and soaked overnight in Milli-Q ultrapure water. Particulates were collected in a 300 μm sieve, covered, and dried overnight in a 60 °C oven.

Irradiations were conducted in a solar simulator, approximating natural sunlight. The system consisted of ten UVA-340+ bulbs (Q-Lab) simulating sunlight in the region of 295 to 365 nm with peak emission at 340 nm. This wavelength range is responsible for the majority of photochemical reactions occurring with environmental plastics debris.^49,50^ Over a 24-h period, this solar simulator system, previously described,^41^ produces irradiation equivalent to one solar day in surface water at the subtropical ocean gyres. Plastics accumulation is significant in this region,^9^ which intercepts over half of global ultraviolet light that reaches the planet surface.^51,52^ Plastic samples were massed using an analytical balance with 0.001 g precision (Sartorius, SECURA324-1S) into 100 mL quartz round-bottom flasks with a target mass of 0.300 ± 0.002 g PP and exact mass recorded. A total of 50 mL of Milli-Q ultrapure water was added to each flask. Two dark controls were prepared as above in glass bottles and wrapped with heavy duty aluminum foil. Dark controls were stored in the solar simulator alongside irradiating samples and sampled after 364 days (i.e., at the end of the experiment). Light samples were continuously irradiated for 364 days (actual time), with two samples sacrificed per time point for analysis, one for size distribution measurements, and one for carbon accounting.

### 2.3 Size Distribution Analysis

Samples removed for size distribution analysis were analyzed with a Malvern Mastersizer 3000 particle sizer (Malvern Analytical) with an attached automated wet dispersion unit. To ensure an appropriate suspension and entrapment of PP particles, the dispersion unit was filled with a 52% methanol–water solution. The irradiated sample was diluted with methanol to reach 52% methanol in water prior to adding the sample to the dispersion unit without any prefiltering. The refractive index was set to 1.36. The measurement method described above was validated with a set of PP particles with sizes ranging from 125 to 710 μm. Based upon the instrument’s manual, the Mastersizer has a maximum measurement range of 0.01 to 3500 μm. Replicate measurements of each sample were taken (*n* ≥ 4) and averaged for analysis; any measurements below the obscuration threshold were excluded.

### 2.4 Carbon Accounting

Samples removed for carbon accounting were vacuum filtered through 0.7 μm pore size and 47 mm diameter Whatman GF/F glass microfiber filters. Filtrate was removed for DOC analysis prior to rinsing the irradiation flask with Milli-Q ultrapure water to attempt to collect all particulates. Filtered particulates were dried overnight at 60 °C and massed on an analytical balance with 0.001 g precision (Sartorius, SECURA324-1S). Plastic recovery had an error of mass of ±0.004 g, n = 10.

Elemental analysis was used to determine the percent carbon composition of PP particulates.^41^ Randomly selected particles of PP from each time point were selected by inserting a metal scoop into the particles and shaking off any excess. Approximately 200–300 μg of particles were packed into 3 × 5 mm tin capsules (Elemental Microanalysis, Marlton, NJ, USA). Samples were massed on an analytical balance with 0.002 mg precision (Sartorius, SECURA26-1S). Capsules were combusted in a Flash 2000 Elemental Analyzer (Thermo Scientific) coupled to a Delta V *Plus* isotope ratio mass spectrometer (Thermo Scientific) calibrated daily with chitin standards to correlate peak area with carbon mass. The average percentage carbon by mass of PP and the gravimetric mass of PP, with plastic-mass recovery determined using the preirradiation mass of each sample recorded at the beginning of the experiment, were used to calculate the mass of plastic-C at each time point.

To measure DOC, filtrate was acidified to pH 2 using hydrochloric acid and analyzed on a Shimadzu TOC-L total organic carbon analyzer.^53^ Certified DOC standard deep seawater reference material available from Consensus Reference Materials (University of Miami) were included in each run and deep sweater reference values verified against the consensus range of 43 to 45 μM. Routine minimum DOC detection limits for the calibration range and instrument configuration are 10 to 15 μM. High concentrations, time point 181 days and later, were diluted prior to DOC measurement.

Carbon accounting was conducted based upon the measurements of DOC and particulate organic carbon, with missing carbon assigned as purgeable carbon based on the following considerations. Particulate carbon above 0.7 μm was collected on GF/F and was plastics-only, given the lack of other carbon sources in the experiments. For DOC, the operational definition was the nonpurgeable organic carbon that passed the 0.7 μm GF/F filters. This may include potential nanoparticles smaller than 0.7 μm but is the community operational definition of DOC.^53^ Finally, all carbon unaccounted for as DOC or particulates was described as purgeable carbon, which includes any volatile organic carbon and inorganic carbon that passes the 0.7 μm filter but is removed by the process of sample acidification and purging.

Time point 53 day particulates were lost during the drying step; therefore, day 53 is not present in particulate or purgeable carbon data.

### 2.5 Assessment of Procedural Controls

Ten procedural controls equivalent to time = 0 days were processed following sampling protocols to determine procedural error. Procedural controls showed minimal error in particulate mass recovery (100.00 ± 0.47%), and the sample handling procedure was deemed suitable to separate POC and DOC. Samples consisting of Milli-Q ultrapure water were analyzed for DOC to evaluate background carbon from water during sample preparation, which was found negligible (∼10 μM).

### 2.6 Scanning Electron Microscopy (SEM) and Energy Dispersive Spectroscopy (EDS)

Randomly selected PP particles were deposited onto carbon adhesive by inverting a vial of particulates over the adhesive; then adhesives were fixed to an aluminum sample holder, and unattached particles were removed using compressed air. Samples were then sputter coated with platinum using a Cressington 108Auto instrument operated at 0.1 mbar vacuum with argon gas. Images were taken on a Hitachi S-4800 scanning electron microscope at 3.0 kV. Energy dispersive spectroscopy was conducted using an attached EDAX system to evaluate fiber contamination (see SI).

### 2.7 Statistical Analysis

Statistical analysis was conducted in JMP and Origin Pro.

## 3 Results and Discussion

### 3.1 Plastic Stability in the Dark

Plastics appeared stable in the dark with no significant difference between plastic masses recovered from the 364-day dark control versus those recovered at day 0 (i.e., the start of the experiment; Table 1). To evaluate the impact of photoirradiation on PP, the plastic-mass and plastic-C recovery of the unirradiated day 0 were treated as 100% and day 0 DOC treated as 0%. Compared to day 0, 99.3 ± 0.5% of the dark control was recovered on day 364, and DOC accumulation in the dark was 0.00 ± 0.00%. The PP particle size of the 364-day dark control was within error of the day 0 sample size, and no discernible structural differences were observed on the particle surface between the two samples in SEM images (see Sections 3.6 and 3.7 for more details). Finally, the percent carbon by mass of the plastic particles did not change in the dark (day 0, 90.4 ± 0.7; dark day 364, 90.1 ± 0.2; Table 1). These results are consistent with other studies reporting the stability of PP in the dark^41,54^ and the insolubility of PP in water.^55^ Thus, biorefractory plastics such as PP are expected to be stable in tranquil aphotic water at ambient temperature.

**Table 1:** Summary of Polypropylene Microplastic Photochemical Degradation, Carbon Tracking, and Chemical Changes Initial, Final, and Percent Recovery of Plastic Mass, as well as Carbon Accounting of Plastic C as Plastic, DOC, or Purgeable Carbon^a^

	Plastic mass recovery			Carbon accounting				
Irradiation time (days)	Starting PP mass (g)	Recovered PP mass (g)	% of initial PP mass recovered	PP % C by mass	% of initial plastic-C recovered	DOC accumulation (mg DOC g plastic-C^–1^)	% of initial plastic-C as DOC	% of initial plastic-C as purgeable-C
0	0.300 ± 0.001	0.296 ± 0.001	100.0 ± 0.5	90.4 ± 0.7	100.0 ± 1.0	0.114 ± 0.022	0.00 ± 0.00	0.00 ± 0.96
26	0.302 ± 0.001	0.292 ± 0.001	98.0 ± 0.5	88.4 ± 1.7	95.6 ± 1.8	0.89 ± 0.18	0.08 ± 0.02	3.98 ± 1.84
53	0.299 ± 0.001	N/A	N/A	N/A	N/A	8.36 ± 0.18	0.82 ± 0.02	N/A
181	0.299 ± 0.001	0.253 ± 0.001	85.9 ± 0.4	86.8 ± 0.5	82.6 ± 0.2	82.09 ± 0.32	8.20 ± 0.03	9.17 ± 0.7
279	0.299 ± 0.001	0.233 ± 0.001	79.3 ± 0.4	85.8 ± 1.4	75.3 ± 1.3	123.0 ± 6.7	12.3 ± 0.67	12.4 ± 1.5
364	0.302 ± 0.001	0.215 ± 0.001	72.5 ± 0.4	85.9 ± 0.8	69.0 ± 0.8	172.9 ± 2.2	17.3 ± 0.22	13.7 ± 0.8
Dark 364	0.301 ± 0.001	0.295 ± 0.001	99.3 ± 0.5	90.1 ± 0.2	99.1 ± 0.6	0.129 ± 0.022	0.00 ± 0.00	0.92 ± 0.57

a Plastic carbon content and DOC accumulation values included for carbon tracking. Missing data for day 56 is due to sample loss during analysis.

### 3.2 Photochemical Loss of Plastic-Mass and Plastic-C

**Figure 1:**
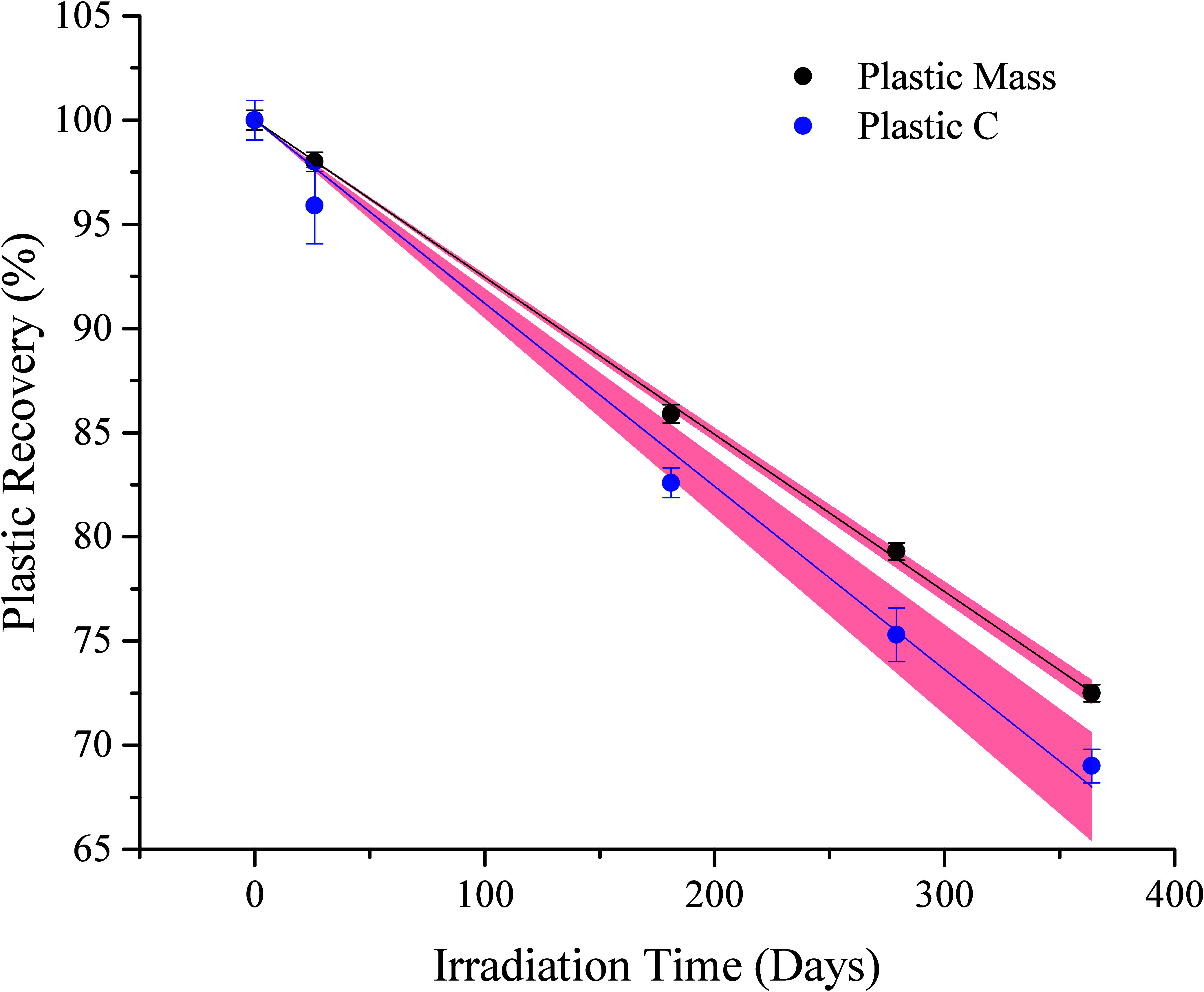
Percent recovery of polypropylene microplastics recovered on 0.7 um GF/F filters after floating on ultrapure water during one year of irradiation, shown as plastic-mass (linear, R^2^ = 0.9999, Table S1) and plastic-C (linear, R^2^ = 0.9998, Table S1). Tjhe shaded region represents 95% confidence interval.

In contrast to the results in the dark, PP microplastics were degraded in light by all metrics (Table 1). Plastic gravimetric mass decreased linearly during irradiation (R^2^ = 0.9999) losing 0.0752 ± 0.0009% per day with 72.5 ± 0.6% recovered after 364 days (Figure 1). Plastic-C mass also decreased linearly (R^2^ = 0.9998, Figure 1). The loss rate of plastic-C was 0.0837 ± 0.0034% per day, and 69.0 ± 0.8% of plastic-C was recovered as particulates on day 364. If linear kinetics are maintained, the rates suggest it would take between 1195 (by plastic-C) and 1330 (by mass) days to completely dissolve the ∼0.5 mm PP microplastics under the conditions of this study (aphotic, ultrapure water, with constant irradiation), equivalent to a lifespan of 3.3 to 3.6 years. Effects of diurnal sunlight exposure were not investigated in this study. We previously reported a comparable rate of 0.0638% mass loss per day for irradiated PP of larger size when floating on seawater (3.45% over 54 days for particles initially ∼3 mm in size) and higher rates of carbon loss than mass loss.^41^

Lifespan estimates of plastics photodegraded under simulated conditions vary widely; Zhu et al. reported a lifespan of 4.3 years for ∼3 mm PP under near identical conditions^41^ and Delre et al. predicted a lifespan of several decades for larger, 6 mm PP disks 1 mm thick.^40^ Variation in lifespans calculated from plastic mass loss may be due to the multitude of factors that influence the rate of photodegradation of a given plastic sample, including surface area to volume ratio (related to particle size and shape), composition of additives, and potential copolymer formulations. Experimental or environmental conditions will also impact the rate of degradation,^56−58^ which is further discussed in Section 3.4.

### 3.3 Kinetics of DOC Photoaccumulation

**Figure 2:**
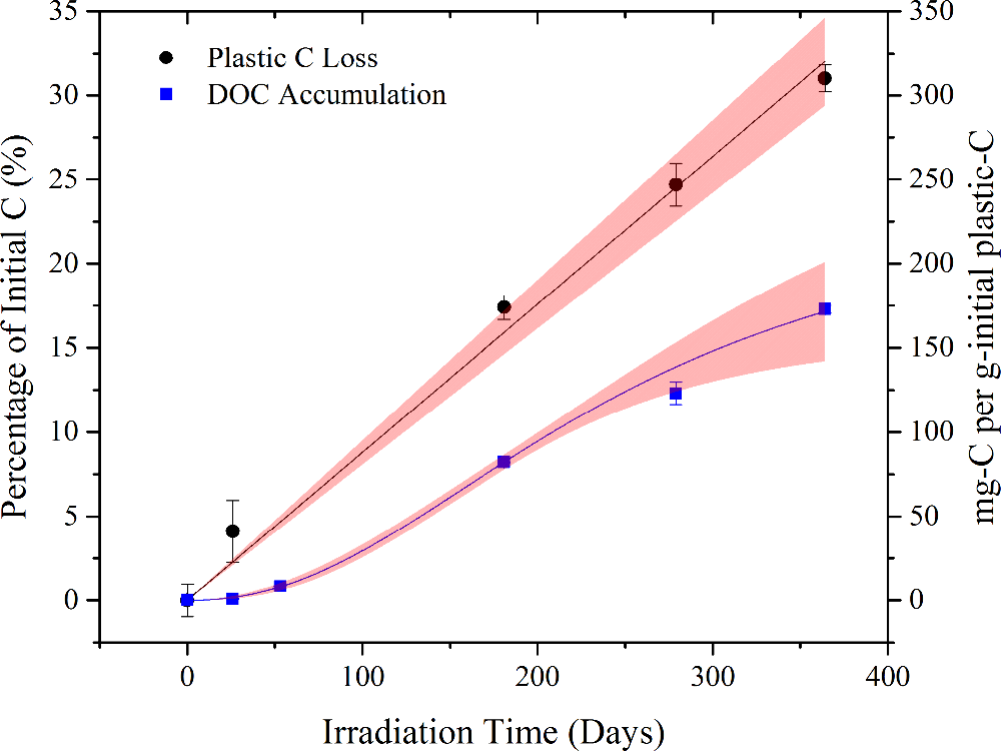
Loss of polypropylene carbon mass (plastic C loss) and dissolved organic carbon (DOC) accumulation, during one year of constant irradiation of polypropylene microplastics floating on ultrapure water displayed as percent of initial plastic C mass (left axis) and as mg-C per g of initial C (right axis). Lines of fit and 95% confidence intervals (shaded regions) are included for plastic C loss (linear, R^2^ = 0.9965, Table S1) and DOC accumulation (sigmoidal, R^2^ = 0.9997, Table S2).

Although plastic-C loss was linear (Figure 2), DOC accumulation was nonlinear and was lower throughout the 364 days than that of the loss of plastic-C (Figure 2). An exponential increase in DOC accumulation was observed through the first 181 days of irradiation and has been previously reported during irradiations of less than one year of PP and other plastics.^41,59^ If our experiments had stopped after half a year, data would also have been best fit by an exponential increase in DOC. However, irradiating samples for one year revealed that plastic-derived DOC accumulation slowed in the second half of the year and that longer term kinetics were best described using a sigmoidal fit (Figure 2; see Table S2 for a comparison of linear, exponential, and sigmoidal fits to the data).

In earlier work, we suggested that the exponential kinetics of DOC accumulation may be used to estimate plastic photodissolution rates.^41^ However, the new longer-term irradiation data presented here indicate that exponential kinetics do not apply to DOC accumulation over longer irradiation times (Figure 2). Using exponentially increasing rates of plastic photoloss based on DOC accumulation results in much shorter lifetimes of plastics in surface waters than when linear rates of plastic loss are applied. For instance, we previously reported photochemical lifetimes for PP ranging from approximately three months based on exponential DOC production kinetics versus 4.3 years based on linear extrapolation of a single measurement of PP mass loss after 54-days of irradiation.^41^ Similar ranges were also reported for PE (6 months using exponential kinetics of DOC production; 33 to 49 years using linear kinetics of mass loss) and expanded polystyrene (∼3 months using exponential kinetics of DOC production; 2.7 years using linear kinetics of mass loss).^41^ As the results presented in this study suggest that linear plastic-mass and plastic-C kinetics should be used, the photochemical lifetimes of microplastics at the sea surface are likely at the longer end of the estimates provided by Zhu et al. (2020).

### 3.4 A Full Accounting of Plastic-C Fate during Photodegradation

To do a full accounting of the apparent fate of plastic-C during irradiation, the percentage DOC accumulation and plastic-C losses were summed. The sum of these two measured terms did not account for all of the plastic-C added at the start of the experiments, even when corrected for procedural controls (i.e., the DOC blanks and the plastic sampling error). Plastic-C was measured as solids captured by a GF/F filter (0.7 μm) and DOC as the nonpurgeable organic carbon that passed through that same filter. Thus, the unaccounted for carbon can be best described as purgeable carbon and could include dissolved inorganic carbon (i.e., dissolved carbon dioxide or carbonates),^60^ and other short chain hydrocarbons, carbonyls, and low molecular weight volatile organic products^8,61^ that can be formed during photodegradation of plastics.

**Figure 3:**
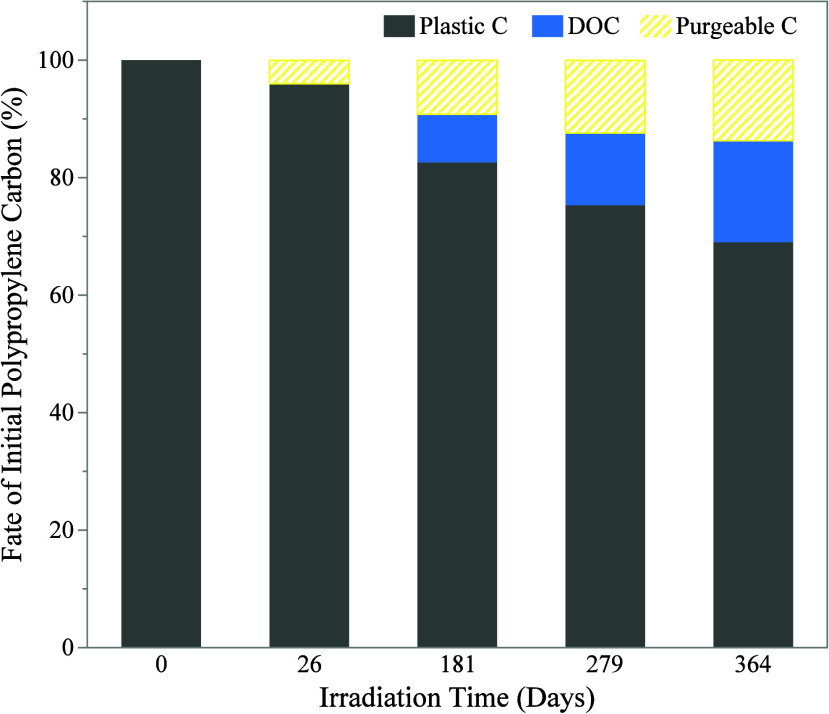
Fate of polypropylene microplastic carbon during one year of constant irradiation floating on ultrapure water under a solar simulator. Data presented as the percentage of the initial plastic carbon recovered as (a) plastic particulates larger than 0.7 μm (Plastic C), (b) dissolved organic carbon smaller than 0.7 μm (DOC), and (c) carbon not accounted for in either the plastic C or DOC pools (Purgeable C). Error bars omitted for clarity. Standard deviations available in Table 1. Data compared to day 0 recovery.

At the start of the experiment, when DOC accumulation was slowest, the majority of carbon lost from the microplastics was purgeable carbon (Figure 3). As DOC accumulation accelerated, the proportion of lost plastic-C accumulating as DOC increased, but the percentage of carbon accumulating as DOC only exceeded purgeable carbon (i.e., accounted for more than 50% of plastic-C losses) on day 364 (Table 1). The existence of a purgeable fraction of C that accounts for the majority of plastic-C losses at most time points also indicates that DOC accumulation will underestimate the plastic particle lifespan. As discussed above (Section 3.3), DOC accumulation had a sigmoidal trend that did not correlate with linear plastic-C loss. Thus, the initial loss of plastic-C may be due mainly to direct production of purgeable forms of carbon (e.g., low molecular weight organics and carbonaceous gases). Alternatively, there may also be initial production of photochemically reactive DOC that is rapidly converted to purgeable carbon in the optically thin water (high UV permeation). In this hypothesis, the gradual accumulation of UV-absorbing compounds in the water may result in increased shielding^62^ and greater DOC accumulation at later time points. The impact of UV-shielding may also be increased due to the experimental design, in which there is no circulation of the water that could diffuse shielding compounds, nor any microbial activity that could remove shielding compounds and is known to consume the DOC formed as plastics photodegrade.^41^ Thus, extrapolation of this hypothesis to natural waters is difficult. While other variables will likely contribute to the trend, over the course of the experiment it can be generally stated that DOC accumulation is affected by the competing factors of DOC production and loss via conversion to purgeable carbon.^63−65^ Toward the end of the one year irradiation, the rate of DOC accumulation slows or halts, likely due to similar rates of DOC production and photochemical losses of DOC. Increased rates of photochemical losses may be conceptualized with simple kinetics whereby the general pool of DOC (in actuality a diverse mix of compounds; Dittmar and Stubbins 2014) is the sole kinetically relevant reactant for the conversion of DOC to purgeable carbon and increased DOC concentration results in increased rates of photochemical losses. Presumably if the experiment were run until all plastic-C is lost, DOC concentrations would decrease beyond the asymptote of the one year kinetics, to a second, final asymptote representing the photoresistant fraction of DOC formed as the plastics photodissolve.

Other kinetic scenarios may also explain the initial slow rates of DOC accumulation and subsequent increase in DOC accumulation rate (autoacceleration), including autocatalysis or autoinduction mechanisms.^66^ Linear polyolefins (i.e., PE and PP) display autocatalytic kinetics during formation of volatiles in thermal degradation through chain scission,^67^ suggesting autocatalytic kinetics may also appear during photodegradation of nanoplastics or short polymer chains in the DOC fraction, as chain scission is also a dominant reaction mechanism.^8^ Autoacceleration has also been observed in production of oxidative products of PP during photoirradiation.^60^ To fully understand the photodegradation of plastic debris in water, more work is needed to deconvolute the kinetics and mechanisms of plastic-C conversion to DOC and plastic-derived DOC photodegradation.

Irradiation in Milli-Q water, which contains only trace levels of ions and nonplastic derived organic carbon, versus in environmental matrices such as seawater, is also expected to impact the mechanisms and rates of photodegradation, complicating comparisons between studies. Dissolved compounds, such as inorganic ions and DOC, are known to impact rates and mechanisms of photodegradation. For example, chlorine ions have been reported to inhibit oxidative aging of PP particles due to quenching of reactive oxygen containing radicals by chlorine radicals.^54^ Conversely, DOC can facilitate indirect photochemistry that increases the overall rate of plastic degradation.^57^ Interactions between ions and dissolved organic matter can also affect the degradation rates. Increased salinity has been observed to improve adsorption of humic acid, a common dissolved organic compound, onto plastics where humic acid can then produce hydroxide radicals and increase photoaging rates.^68^ Humic acid can also quench chlorine radicals thereby reducing chlorine inhibition of plastic degradation.^69^ The role of ions and other dissolved compounds in determining the plastic photodegradation mechanisms and rate is undeniably important. However, in our previous work irradiating larger (3 mm) PP microplastic under the same light conditions but floating in natural seawater, we determined near-identical rates of plastic mass loss (i.e., 0.0638% per day in Zhu et al. versus 0.0752 ± 0.0009% per day here for 579 ± 12 μm PP microplastics; Figure 1). Thus, although changes in the salt content of natural waters can impact rates and mechanisms of microplastic photodegradation, the effects appear modest.

### 3.5 Photochemical Changes in the Carbon Content of Microplastics

The carbon content of the PP microplastics decreased from 90.4 ± 0.7% C by mass on day 0 to 85.9 ± 0.8% C by mass on day 364 (Table 1). Most of the decrease in percent carbon content by mass occurred within the first 26 days of the experiment, with slight but insignificant reductions occurring from day 26 through day 364 (one-way ANOVA, p = 0.3609, α = 0.05). Photochemical degradation of plastics is known to introduce oxygen containing functional groups such as carbonyls, lactones, peroxides, and esters.^8,70^ Studies show a rapid increase in oxygenation of PP can begin in as few as 2 days of irradiation^71^ with evidence of autoacceleration during the first 25 days of irradiation.^60^ Oxygen content as measured by indicators of oxygen-containing functional groups (carbonyl index, chemiluminescence of hydroperoxides, etc.) gradually stabilizes during long irradiations (i.e., beyond 25 days).^60,71^ These trends in oxygen uptake may explain the initial decrease in carbon content during the first 26 days of this study and following stabilization of carbon content from day 26 onward.

### 3.6 Photochemical Changes to Microplastic Surface Structure

**Figure 4:**
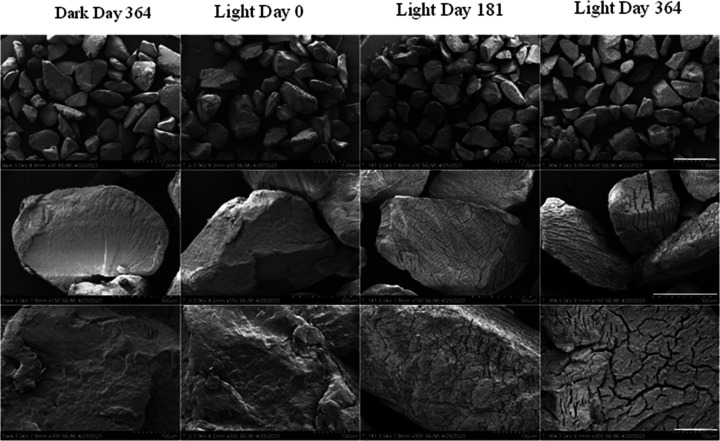
Scanning electron microscopy images of polypropylene particles (left to right: dark control for day 364, unirradiated day 0, irradiated day 181, irradiated day 364). Full scale bars top to bottom: 1.00 mm, 300 μm, and 100 μm; a white line above each scale bar in the right column has been added for visibility. Each tick represents 1/10th of full-scale length. Additional time points available in the SI.

The surfaces of unirradiated (day 0), irradiated, and dark control PP microplastics were examined using SEM (Figure 4). At the beginning of the experiment, the microplastics had irregular shapes with both smooth and angled edges, attributed to the production of these particles by cryomilling larger plastic granules (see Section 2.2). Their surfaces were free of pitting and cracking, appearing smooth at low magnification (8.3 mm ×30). At higher magnification (8.2 mm ×150 and ×300), a rough surface was visible with the appearance of directionally organized, scale-like layers. The dark day 364 control was similar to the day 0 sample, with no identifiable differences in particle appearance and surface structure. At high magnification fibers were visible on the surface of some particles. Using energy dispersive spectroscopy (Figure S1), these fibers were identified as glass microfibers that were contaminated from the GF/F filters used to collect the microplastics for SEM imaging.

**Figure 5:**
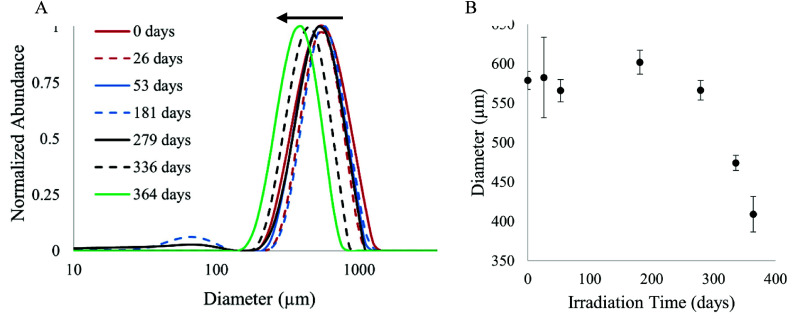
Size distribution of polypropylene microplastics during one year of constant irradiation floating on an ultrapure water under a solar simulator. (A) Size distribution of particles exposed to simulated sunlight for 0 to 364 days. (B) Particle size distribution mode with standard deviation of replicate measurements (*n* ≥ 4 for all time points; mode describes the larger, primary population for time points 181 and 279 with bimodal distribution).

Day 26 irradiated samples were not visually distinguishable from either day 0 or day 364 dark controls. However, from day 181 of irradiation onward, distinct surface cracking was observed on all particles. A visual trend was observed with the width and length of cracks increasing with irradiation time. Two distinct cracking patterns were observed: (1) regions of parallel, predominantly linear cracks with little branching, and (2) regions of unorganized cracking with shorter cracks that may be linear or curved and frequently branch (Figure 4). Some single particles had both cracking patterns. However, each face of a particle displayed only one pattern. Of particle faces visible in the imaging, unorganized cracking appears to predominate. The patterns may indicate regions of direct vs indirect irradiation based on particle orientation at the water surface during irradiation or may be related to regions of differing crystallinity or crystal orientation, which are known to impact physical properties of PP.^72,73^ Alternatively, the cracking may have resulted from the grinding process used to prepare the PP samples and was later exacerbated by irradiation. However, similar cracking as both the organized and unorganized patterns have been reported for polyolefins fragments collected on beaches^74^ suggesting these cracks are not likely an artifact of the sample preparation process. A small number of particles smaller than approximately 150 μm were distinguishable from the starting ∼600 μm PP particles on day 181 (Figure 5), indicating that cracking produced occasional fragments. In agreement with the size distribution data below, the smaller fragments visible via SEM did not constitute a power law increase in counts with decreasing size, nor even a majority of particles in any sample. Care was taken with the samples to avoid all mechanical stress on the particles prior to measurements to avoid fragments from any cause other than irradiation, but it is possible that the small number of fragments visible in images resulted from mechanical forces inherent in sample handling and preparation (e.g., physical manipulation, air pressure, or vacuum).

### 3.7 Impact of Photochemistry on Microplastics Size Distributions

The population of microplastics in the unirradiated day 0 sample was symmetrically unimodal in size with a mode of 579 ± 12 μm (Figure 5). The size distribution of PP microplastics did not change following 364 days of incubation while floating on Milli-Q water in the dark (Table 1). In the light, the size distributions of microplastics remained dominated by a symmetrical unimodal population throughout. However, the mode of this unimodal population decreased throughout the irradiation, reaching 409 ± 22 μm on day 364 (Figure 5). Although most particles fell within the unimodal population, a small second population of particles centered around 67 μm emerged on day 181 when it represented 7% of the particle counts. This second population was also visible on day 279, though at a lower abundance (6% of counts) and a greater span than on day 181. This secondary population disappeared for the remaining irradiation time points, and its cause is unknown. The distribution of particle sizes in the samples was evaluated through peak span (describing the range of particle sizes) and calculated using the 10, 50, and 90 percentiles of particle counts. Peak spans ranged from 0.75 to 1.02 with no statistically significant correlation between span and irradiation time (Table S3).

When considering the above results, it is important to remember the analytical fractions that are being addressed. The Mastersizer used to count particles was calibrated with PP down to 125 μm and had a minimum detection limit of 0.01 μm. Thus, if nanoplastics were produced below 0.01 μm, they would not be identified. Photoproduction of nanoplastics in the sub-0.5 μm range have been reported during the photoirradiation of polystyrene^75^ but fall within the typical category of DOC operationally defined as submicron (typically <0.7 μm or <0.45 μm).^76^ In this work, any sub-0.7 μm nanoparticles produced would be captured in the DOC fraction when accounting for carbon in the system, and consideration of fragmentation is limited to microplastics.

Mechanical fragmentation results in increasing particle abundance with decreasing particle size via a power relationship.^18,77^ Field studies of microplastics in natural waters have reported that particle abundance follows a power function (i.e., mechanical fragmentation pattern) with increasing particle numbers as size decreases.^19−28^ Based on our results, photodegradation of PP microplastics while floating on tranquil Milli-Q water did not result in size distributions consistent with photofragmentation being a dominant process; instead, results suggest that photochemical losses occurred from the surface of microplastics, indicated by plastic-mass loss and DOC accumulation, was the main driver of changing size distributions. In this case, particle size distributions depended on the initial size and abundance of particles added, and particle count should remain constant throughout the irradiation with the particles shrinking in size as they lose mass and carbon.

Multiple studies have demonstrated a link between photoirradiation and increased brittleness.^78,79^ The extensive cracking observed via SEM imaging at the surface of the plastic in this and other studies likely contributes to changes in tensile strength in plastics after irradiation.^7,80,81^ Cracking and brittleness can also explain the link between photoirradiation and subsequent formation of a greater number of nano- and microscale fragments in response to mechanical forces, such as stirring or abrasion with sand.^15,16,82,83^ The presence of water may also be critical in determining which mechanism of degradation will predominate during photoirradiation, as a recent study by Song et al. demonstrated particles, including large numbers of nanoparticles, were generated from PP irradiated under dry conditions without mechanical force.^14^ Our results complement these findings, demonstrating that sunlight exposure without mechanical force will predominantly result in carbon conversion to DOC and/or purgeable forms of C without significant particle production when PP is irradiated floating in water. When irradiation is combined with mechanical forces, these past studies have shown that fragments are produced in the expected power-law relationship with increased abundance as size decreases. Thus, photoirradiation likely enhances fragmentation of plastic debris in surface water environments experiencing mechanical forces like wave action, abrasion with sand, or impacts, resulting in a power-law relationship between size and abundance as reported in field studies.^15,16,22−24,82^ However, models suggest that particles in the low μm range will not experience sufficient stress from wave action to promote fragmentation and in these cases dissolution may predominate.^84^

The results of this study show that for sub-millimeter PP sunlight exposure induced photodissolution without significant fragmentation over one year of irradiation. Instead of fragmenting into a greater number of smaller microplastics, the initial population of microplastics shrank in size. The conversion of plastic-C to DOC or purgeable carbon due to photoirradiation is a powerful route of sub-millimeter plastic removal. In this work, we demonstrate that photodissolution of PP floating in calm water can occur without production of secondary fragments.

## Supporting Information Available

Tables describing fit equations for all figures in the main text; DOC accumulation fits and tabulated size distribution peak spans; energy dispersive spectroscopy of glass microfiber contamination in SEM images; and additional time points of SEM images not included in the main text for clarity (PDF)
