# Supplementary material for: Sunlight-Driven Photochemical Removal of Polypropylene Microplastics from Surface Waters Follows Linear Kinetics and Does Not Result in Fragmentation
Source: Environ Sci Technol. 2024 Mar 15;58(12):5461–71. doi: 10.1021/acs.est.3c07161 (PMC10976886; doi:10.1021/acs.est.3c07161)
Supplement: Supplementary file 1 — es3c07161_si_001.pdf [file es3c07161_si_001.pdf]

Supporting Information

Sunlight-driven photochemical removal of polypropylene microplastics from surface waters follows linear kinetics and does not result in fragmentation

Erin Tuttle*[1], Charlotte Wiman[2], Samuel Muñoz[2,3], Kara Lavender Law[4], and Aron Stubbins[2,3,5]

[1]Department of Biological and Physical Sciences, Assumption University, Worcester, MA, 01609, USA.
[2]Department of Marine and Environmental Science, Northeastern University, Boston, MA, 02115, USA. [3]Department of Civil and Environmental Engineering, Northeastern University, Boston, MA, 02115, USA.
[4]Sea Education Association, Woods Hole, MA, 02540, USA
[5]Department of Chemistry and Chemical Biology, Northeastern University, Boston, MA, 02115, USA.
*Corresponding author, ee.tuttle@assumption.edu

List of Contents

4 pages:

Table S1 – Linear fit equations for percent plastic-mass and plastic-C recovery and percent-C loss of polypropylene over one year of irradiation

Table S2 – Comparison of fit equations for DOC accumulation during irradiation of polypropylene over one year

Table S3 – Peak spans for size distributions of timepoints

Figure S1 – Energy dispersive X-ray spectrum of glass microfiber contamination

Figure S2 – Scanning electron microscopy images for additional timepoints

Supplemental Information

Table S1: Linear fit equations for percent plastic-mass and plastic-C recovery and percent-C loss of polypropylene over one year of irradiation. Fits constrained to 100 at 0 days for recoveries and 0 at 0 days for loss, matching experimental conditions.

$$y = mx + b$$

	m (%)	b (%)
Plastic-mass recovery	-0.07544±0.00057	100
Plastic-C recovery	-0.08791±0.00259	100
Plastic-C loss	0.08791±0.00259	0

Table S2: Comparison of models of fit for dissolved organic carbon (DOC) accumulation. mg DOC accumulation per g initial plastic-C abbreviated as mg-DOC*gC^{-1}.

Model Type	Function	Reduced Chi-Sqr	R^2
Linear	Y = mX +b<br>m = -11.1±6.4 mg-DOC*gC^{-1}*days-1<br>b = 0.517±0.033 mg-DOC*gC^{-1}	106.8	0.9907
Exponential	$Y = y0 + a*e^{cX}$<br>y0 = -180±154 mg-DOC*gC^{-1}<br>a = 164±150<br>c = 0.00216±0.00141 days^{-1}	79.6	0.9911
Sigmoidal	$y = A2 + (A1 - A2)/(1 + (x/x0))^p$<br>A1 = 0.108±0.065 mg-DOC*gC^{-1}<br>A2 = 235±20 mg-DOC*gC^{-1}<br>$x0$ = 137±15 days<br>p = 2.31±0.08	10.7	0.9997

Table S3: Peak spans from size distribution analysis of irradiated polypropylene, averaged from D10 and D90 values (n≥4).

Irradiation Time (Days)	Peak Span (average, n≥4)
0	1.02
26	0.75
53	0.89
181	0.92
279	0.98
336	0.81
364	0.81

A

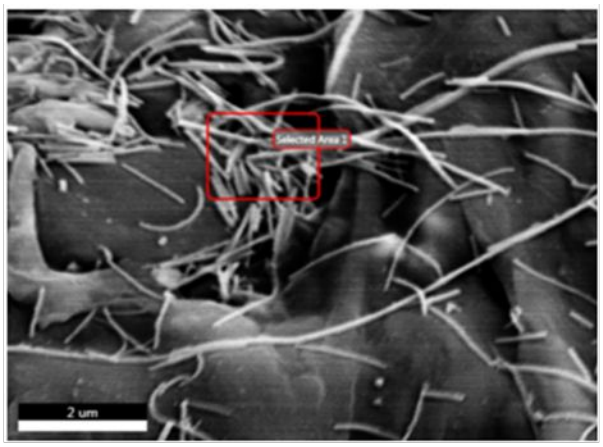

B

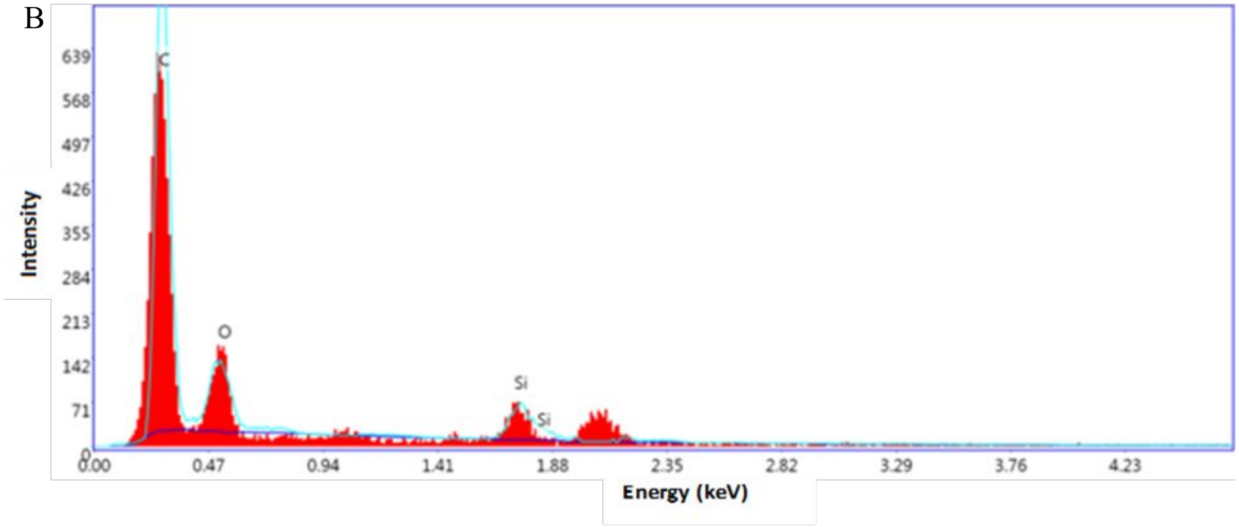

Figure S1: Energy dispersive spectroscopy analysis of microfiber contamination on polypropylene particles. Image area selected for high density of the fibers. a) scanning electron microscopy image of selected region, b) EDS spectrum.

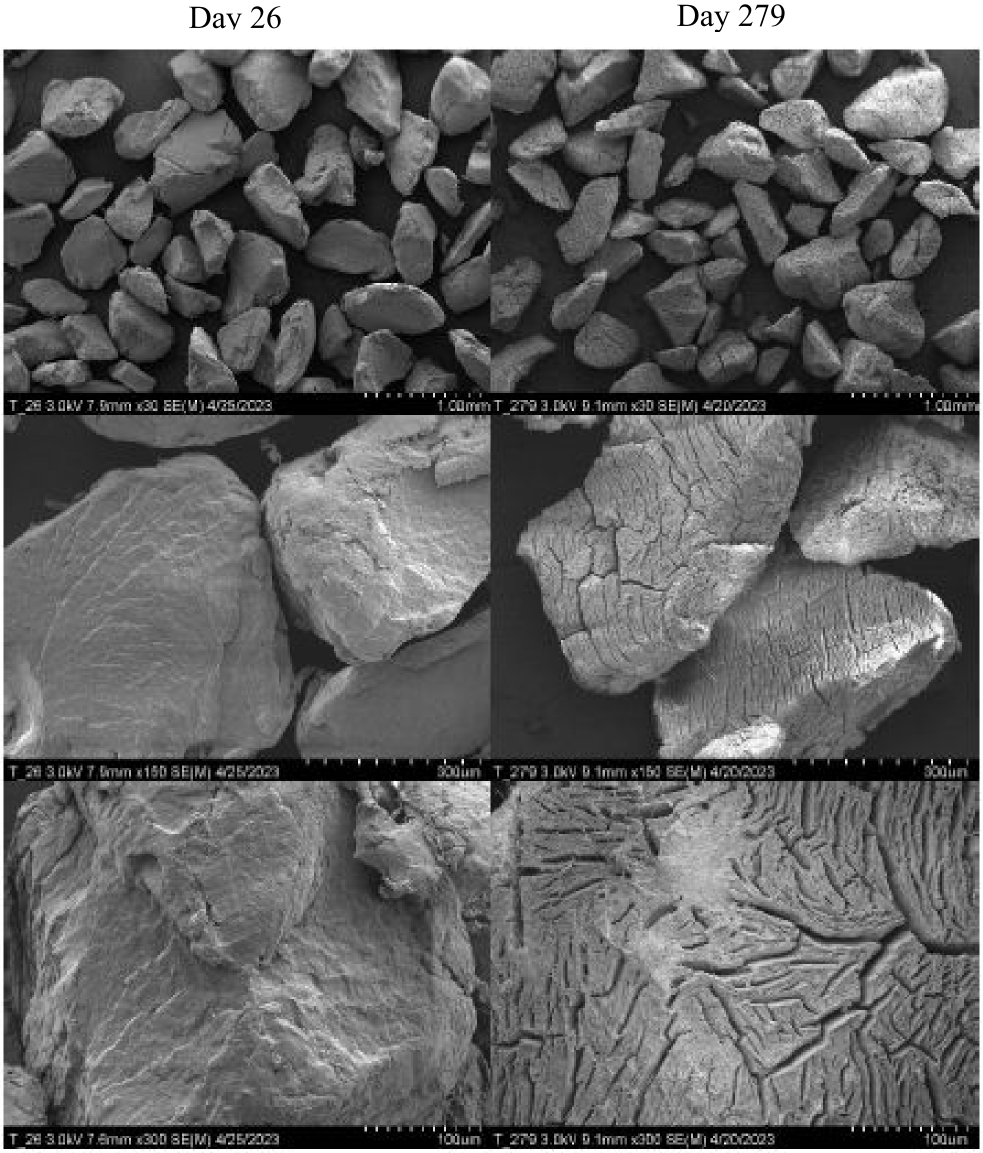

Figure S2: Scanning electron microscopy images of polypropylene particles at additional timepoints (left to right irradiated day 26, irradiated day 279). Full scale bars top to bottom: 1.00 mm, 300 µm, and 100 µm. Each tick represents 1/10th of full-scale length. Additional timepoints in main manuscript.
